# A cohort study of 676 patients indicates D-dimer is a critical risk factor for the mortality of COVID-19

**DOI:** 10.1371/journal.pone.0242045

**Published:** 2020-11-09

**Authors:** Yongsheng Huang, Xiaoyu Lyu, Dan Li, Lin Wang, Yujun Wang, Wenbin Zou, Yingxin Wei, Xiaowei Wu

**Affiliations:** 1 School of Basic Medicine, Peking Union Medical College, Institute of Basic Medical Sciences, Chinese Academy of Medical Sciences, Beijing, China; 2 Department of Endocrinology, the Central Hospital of Wuhan, Tongji Medical College, Huazhong University of Science and Technology, Wuhan, China; 3 National Cancer Center/ Cancer Hospital, Chinese Academy of Medical Sciences and Peking Union Medical College, Beijing, China; 4 Department of Critical care medicine, the Central Hospital of Wuhan, Tongji Medical College, Huazhong University of Science and Technology, Wuhan, China; 5 Department of Thoracic Surgery, TongJi Hospital, TongJi Medical College, Huazhong University of Science and Technology, Wuhan, China; 6 Department of General Surgery, Peking Union Medical College Hospital, Chinese Academy of Medical Sciences and Peking Union Medical College, Beijing, China; Mahidol Oxford Clinical Research Unitl (MORU), THAILAND

## Abstract

Coronavirus Disease 2019 (COVID-19) has recently become a public emergency and a worldwide pandemic. However, the information on the risk factors associated with the mortality of COVID-19 and of their prognostic potential is limited. In this retrospective study, the clinical characteristics, treatment and outcome data were collected and analyzed from 676 COVID-19 patients stratified into 140 non-survivors and 536 survivors. We found that the levels of Dimerized plasmin fragment D (D-dimer), C-reactive protein (CRP), lactate dehydrogenase (LDH), procalcitonin (PCT) were significantly higher in non-survivals on admission (non-survivors vs. survivors: D-Dimer ≥ 0.5 mg/L, 83.2% vs. 44.9%, *P<*0.01; CRP ≥10 mg/L, 50.4% vs. 6.0%, *P<*0.01; LDH ≥ 250 U/L, 73.8% vs. 20.1%, *P<*0.01; PCT ≥ 0.5 ng/ml, 27.7% vs. 1.8%, *P<*0.01). Moreover, dynamic tracking showed D-dimer kept increasing in non-survivors, while CRP, LDH and PCT remained relatively stable after admission. D-dimer has the highest C-index to predict in-hospital mortality, and patients with D-dimer levels ≥0.5 mg/L had a higher incidence of mortality (Hazard Ratio: 4.39, *P*<0.01). Our study suggested D-dimer could be a potent marker to predict the mortality of COVID-19, which may be helpful for the management of patients.

## 1. Introduction

In December 2019, a novel coronavirus disease (COVID-19) caused by Severe Acute Respiratory Syndrome Coronavirus 2 (SARS-CoV-2) infection broke out in Wuhan and spread rapidly throughout China. As of October 14th, 2020, World Health Organization (WHO) reported 38,002,699 COVID-19 cases have been confirmed in the world, and 1,083,234 people died from COVID-19 [1]. Human to human transmission has accounted for most if not all of the COVID-19 infections, including the medical personnel in hospital [2, 3]. The clinical manifestations of COVID-19 included fever, cough, diarrhea, dyspnea, fatigue and pneumonia [4–6]. Most patients with COVID-19 are considered as non-severe patients and the symptoms are generally mild. However, the symptoms in about 10% of COVID-19 patients are severe and some progress rapidly to critical conditions, including organ dysfunction, such as acute respiratory distress syndrome (ARDS), acute cardiac injury, acute kidney injury and even death [7, 8]. Concerns about a possible association between ethnicity and outcome were rising. Evidence is continuing to emerge that the people from black, Asian, and minority ethnic (BAME) communities are susceptible to COVID-19 pandemic [9–11]. To date, only Remdesivir [12–15] and Dexamethasone [16, 17] have demonstrated a positive response in a prospective for the treatment of patients with COVID-19, while Chloroquine or Hydroxychloroquine were suggested to be used with caution because they have a lot of side effects and their efficacy are controversial [18–20]. So far, there is no effective vaccine for COVID-19 used in population on large scale. Besides, the prognosis methods for the mortality of COVID-19 patients were still limited. Thus, our objective of this cohort is to explore the clinical risk factors for rapid predicting the severity and mortality of COVID-19 and put it into practice. Our study would be helpful for setting up different treatment and personal therapy route for COVID-19 patients.

## 2. Methods

### 2.1 Data sources

We performed a retrospective study on 676 laboratory-confirmed cases with COVID-19 based on clinical indications from Wuhan TongJi Hospital and Central Hospital of Wuhan in the Hubei provinces, China, from January 1 through April 6, 2020. We divided COVID-19 patients into survival and non-survival (dead) subgroups according to endpoint. All the confirmed case with COVID-19 was defined as a positive result to real-time reverse-transcriptase polymerase-chain-reaction (RT-PCR) assay using nasal and pharyngeal swab specimens [5]. The clinical characteristics and laboratory assessments were extracted from the available electronic medical records. The ethics committee of Wuhan Tongji Hospital and Central Hospital of Wuhan approved this study and granted a waiver of informed consent in light of the urgent need to collect clinical data.

### 2.2 Radiologic assessments and laboratory assay

Radiologic assessments included chest X-ray and computed tomography (CT). We evaluated and divided the patients into three groups according to the extent of lung lesions: mild (0–30%), moderate (30%-60%) and severe (>60%). There are two authors (W. X. and H. Y.) making the independent evaluation and the statistics. The blood samples of patients were collected within 24 hours after admission, and then consistent collected during the treatment. Laboratory assessments consisted of complete blood count, blood chemistry, coagulation test, liver and renal function, C-reactive protein, lactate dehydrogenase and creatine kinase.

### 2.3 Statistics of the cohort study

Continuous variables were expressed as the means and standard deviations or medians and interquartile ranges (IQR) as appropriate. Categorical variables were summarized as the counts and percentages in each category. Mann-Whitney test and two-tailed t-test and were applied to continuous variables, the frequencies of categorical variables were compared using the chi-square test and Fisher’s exact test as appropriate. The candidate risk factors included age, sex, underlying disease and abnormal laboratory findings. Concordance index (C-index) and its 95% confidential interval (95% CI) were evaluated by receiver operator characteristic (ROC) curve. The Hazard Ratios (HRs) of risk factors were analyzed with Cox-proportional hazard models. We used a backward stepwise selection procedure to interactively and automatically select the optimal smallest subset of variables that can predict the dependent variable to the best extent. This process of course guarantees that variables included in the final model are independent. The outcomes of patients were compared by Kaplan-Meier survival analysis, which were calculated by log rank tests. The tests with *P* value less than 0.05 was considered statistically significant. Statistical analyses were done using the Statistical Package for the Social Sciences (SPSS) software (version 21.0).

## 3. Results

### 3.1 Clinical characteristics of the cohort at the onset of admission

The general clinical characteristics of 676 COVID-19 cases in our study are shown in **Table 1**. Patients were categorized into 140 non-survival and 536 survival subgroups according to endpoint. In our cohort, the median age in the survival population was 51.0 year, interquartile range (IQR) was 31.0 to 64.0, in non-survival population was 67.0 years (IQR: 57.0 to 75.0). The age differed significantly between the two subgroups, and 70.0% of non-survivors were aged above 60 years. The mortality of COVID-19 was higher in men than in women (69.3% vs. 30.7%). Fever (79.7%), cough (66.7%) and fatigue (38.5%) were the most common symptoms, whereas diarrhea (8.1%) was rare. Notably, fever occurred more frequently in the patients in the non-survival group than the survival group (86.4% vs.78.0%). We also found the underlying disease were risk factors associated with the mortality of COVID-19 in our cohort. The non-survivals were more likely had common chronic diseases than survivals, such as hypertension (60.0% vs. 26.5%), diabetes (19.3% vs. 13.6%), heart disease (17.9% vs. 8.6%) and cancer (11.4% vs. 3.2%). In the radiologic assessments, more lung lesions were found in the non-survivals than the survivals (65.9% vs. 16.0%).

**Table 1:** Clinical characteristics of the patients with COVID-19 on the initial admission.

Clinical characteristics		All patients (n = 676)	Subgroups		
			non-survivals (n = 140)	survivals (n = 536)	*P* value
Age, Median (IQR)		56.0 (39.0–68.0)	67.0 (57.0–75.0)	51.0 (36.0–64.0)	<0.01
Age groups -No./total No. (%)					
	<40 yrs	175/676 (25.9%)	5/140 (3.5%)	170/536 (31.7%)	<0.01
	40–60 yrs	208/676 (30.8%)	37/140 (26.4%)	171/536 (31.9%)	0.09
	≥ 60 yrs	293/676 (43.3%)	98/140 (70.0%)	195/536 (36.3%)	<0.01
Female sex -No./total No. (%)		362/676 (53.6%)	43/140 (30.7%)	319/536 (59.5%)	<0.01
Symptoms -No./total No. (%)					
	Fever	539/676 (79.7%)	121/140 (86.4%)	418/536 (78.0%)	0.027
	Cough	451/676 (66.7%)	92/140 (65.7%)	359/536 (67.0%)	0.778
	Fatigue	260/676 (38.5%)	58/140 (41.4%)	202/536 (37.7%)	0.418
	Diarrhea	55/676 (8.1%)	9/140 (6.4%)	46/536 (8.6%)	0.407
**Underlying disease:**					
	Hypertension	226/676 (33.4%)	84/140 (60.0%)	142/536 (26.5%)	<0.01
	Diabetes	100/676 (14.8%)	27/140 (19.3%)	73/536 (13.6%)	0.09
	Heart disease	71/676 (10.5%)	25/140 (17.9%)	46/536 (8.6%)	0.01
	Cancer	33/676 (4.9%)	16/140 (11.4%)	17/536 (3.2%)	<0.01
**Radiologic assessments:**					
	Mild	342/660 (51.8%)	28/129 (21.7%)	314/531 (59.1%)	<0.01
	Moderate	143/660 (21.7%)	16/129 (12.4%)	127/531 (23.9%)	<0.01
	Severe	175/660 (26.5%)	85/129 (65.9%)	90/531 (16.0%)	<0.01
**laboratory findings:**					
Median PaO2:FIO2 ratio		3.4 (2.4–5.1)	1.5 (1.0–2.2)	4.0 (2.8–5.2)	<0.01
WBC, * 10^9/L		5.0 (3.9–6.7)	6.5 (4.5–10.3)	4.87 (3.79–6.20)	0.006
	>10.0	58/676 (8.6%)	39/140 (27.9%)	19/536 (3.5%)	<0.01
	<4.0	184/676 (27.2%)	25/140 (17.9%)	159/536 (29.7%)	<0.01
Neutrophil count, * 10^9/L		3.42 (2.30–4.98)	4.9 (3.2–9.0)	3.18 (2.25–4.49)	<0.01
Lymphocyte count, *10^9/L		1.02 (0.68–1.38)	0.64 (0.44–0.99)	1.09 (0.77–1.45)	<0.01
	< 1.5	524/676 (77.5%)	116/140 (82.9%)	408/536 (76.1%)	0.09
Platelet count, *10^9/L		178.0 (134.0–224.3)	134.0 (107.5–184.5)	182.0 (143.0–231.0)	<0.01
Haemoglobin level, g/dl		128.0(119.8–140.0)	126.0 (112.5–138.5)	129.0 (121.0–140.0)	0.024
D-dimer, mg/L		0.53 (0.25–1.21)	1.55 (0.61–8.88)	0.44 (0.23–0.91)	<0.01
	≥ 0.5	331/636 (52.0%)	99/119 (83.2%)	232/517 (44.9%)	<0.01
CRP, mg/L		2.05 (0.45–5.47)	8.40 (3.80–87.90)	1.33 (0.34–3.93)	<0.01
	≥10	95/646(14.7%)	64/127 (50.4%)	31/519 (6.0%)	<0.01
PCT, ng/ml		0.06 (0.04–0.10)	0.22 (0.08–0.52)	0.05 (0.04–0.08)	<0.01
	≥0.5	42/606 (6.9%)	33/119 (27.7%)	9/487 (1.8%)	<0.01
LDH, U/L		194.0 (153.0–273.0)	377.0 (236.0–512.0)	182.0 (147.0–235.0)	<0.01
	≥250	191/614 (31.1%)	93/126 (73.8%)	98/488 (20.1%)	<0.01
AST, U/L		24.0 (17.5–36.2)	38.0 (28.0–47.1)	21.3 (17.0–32.3)	<0.01
	>40	148/659 (22.5%)	61/125 (48.8%)	87/534 (16.3%)	<0.01
ALT, U/L		21.0 (13.3–33.8)	25.2 (16.2–35.5)	19.7 (13.0–33.7)	0.64
CK, U/L		77.0 (48.0–139.0)	137.7(64.3–264.0)	73.2 (45.9–123.4)	<0.01
Creatinine, umol/L		65.0 (52.0–78.4)	78.3 (60.7–106.2)	63.0 (51.3–75.7)	<0.01
Blood urea nitrogen, mmol/L		4.1 (3.3–5.5)	6.4 (4.6–9.1)	4.0 (3.2–5.0)	<0.01
Total bilirubin, μmol/L		8.8 (6.5–12.2)	11.4 (6.7–15.7)	8.6 (6.4–11.4)	0.25
Interleukin-6, pg/ml		4.8 (2.3–19.3)	14.4 (8.8–49.0)	3.5 (2.2–8.2)	<0.01

Abbreviations: IQR, interquartile range; PaO2:FIO2 ratio: partial pressure of arterial oxygen to the fraction of inspired oxygen; WBC, white blood cell; D-dimer, Dimerized plasmin fragment D; CRP, C-reactive protein; PCT, procalcitonin; LDH, lactate dehydrogenase; AST, aspartate aminotransferase; ALT, alamine aminotransferase; CK, creatinine kinase; *P* values comparing non-survival cases and survival cases are from χ^2^ or Fisher’s exact test.

The results of blood cell test on the initial admission were shown in **Table 1**. Of 676 patients, 77.5% of patients had lymphopenia and the non-survival group is mild likely to develop lymphopenia (non-survivors vs. survivors: 82.9% vs. 76.1%). On the initial admission, cases also had other prominent laboratory abnormalities as compared to the survival cases (**Table 1**), such as neutrophilia, thrombocytopenia, elevated levels of D-dimer (D-Dimer ≥ 0.5 mg/L: 83.2% vs. 44.9%, *P<*0.01), C-reactive protein (CRP ≥10 mg/L: 50.4% vs. 6.0%, *P<*0.01), procalcitonin (PCT ≥ 0.5 ng/ml: 27.7% vs. 1.8%, *P<*0.01), lactate dehydrogenase (LDH ≥ 250 U/L: 73.8% vs. 20.1%, *P<*0.01), and aspartate aminotransferase (AST >40 U/L: 48.8% vs. 16.3%, *P<*0.01).

### 3.2 Dynamics of clinical risk factors in patients with COVID after admission

**Fig 1:**
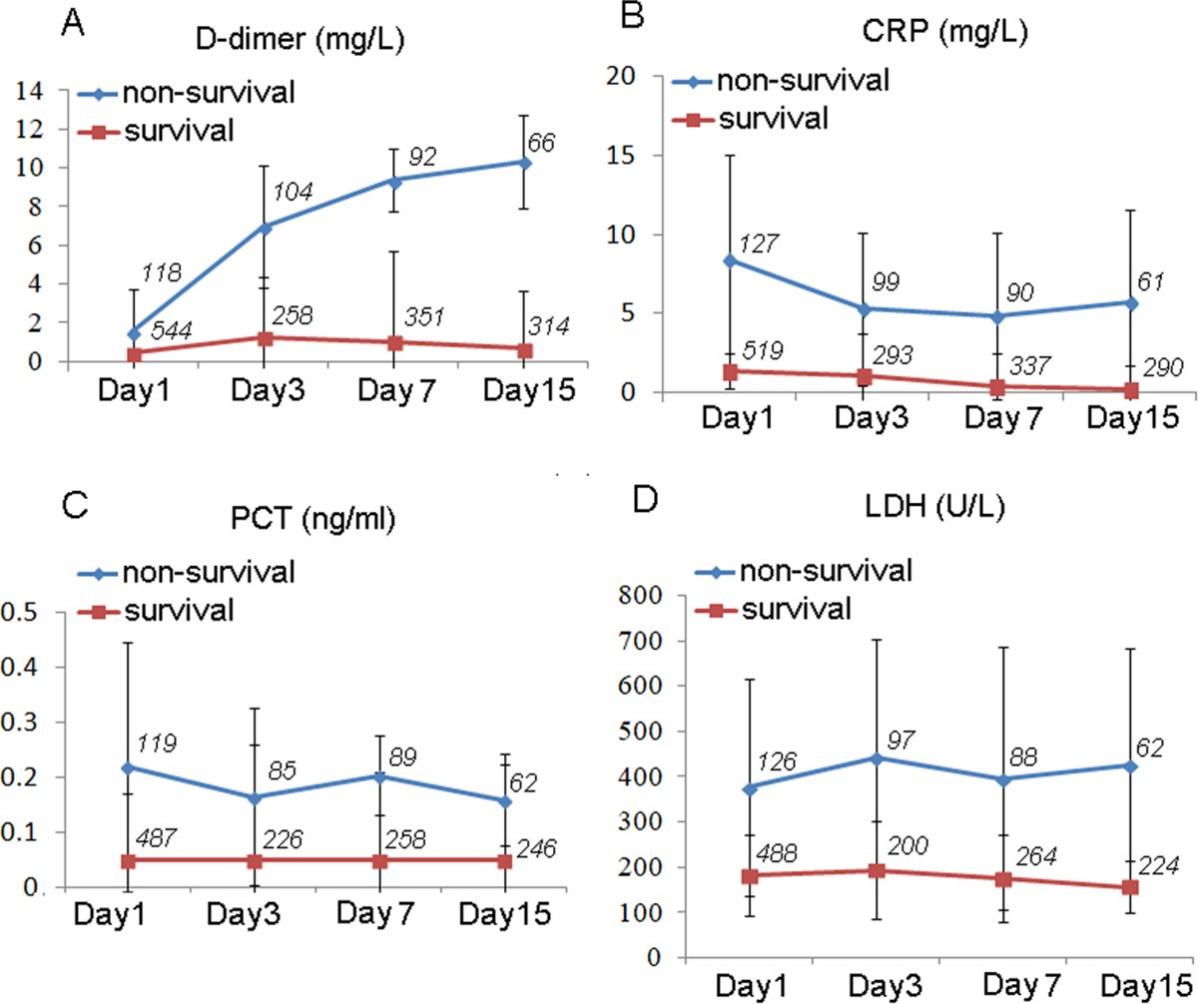
Dynamical changes of the risk factors in the patients with COVID-19 during hospitalization. Figure shows dynamical changes of CRP (A), LDH (B), PCT(C) and D-dimer (D) in patients within 15days from admission onset. We stratified the patients into two subgroups: non-survivals and survivals. The case numbers in the dynamic analysis are shown as *italics* in the pictures. Data represent mean ± SD. Abbreviations: D-dimer, Dimerized plasmin fragment D; CRP, C-reactive protein; LDH, lactate dehydrogenase; PCT, procalcitonin.

In our study, we also evaluated the dynamic changes of clinical risk factors during hospital stay between survival and non-survival COVID-19 patients. Of the clinical risk factors evaluated, we found the levels of D-dimer, CRP, LDH, and PCT are all significantly higher in non-survivors than in survivors at the admission onset (**Fig 1**). Interestingly, we found that D-dimer level still strikingly increased in some non-survivors after admission. On the initial admission, D-dimer levels were significantly higher in the non-survivals than in the survivals [median (mg/L): 1.55 vs. 0.44]. After 15 days admission, the difference of D-dimer levels between the two groups was even greater [median (mg/L): 10.33 vs. 0.95] (**Fig 1**). However, the levels of CRP, LDH, and PCT were relatively consistent in non-survival patient after admission. Since D-dimer originates from the formation and lysis of cross-linked fibrin and reflects activation of coagulation, it was suggested more thrombosis occurred in the non-survivors during the hospital-stay.

### 3.3 High D-dimer levels predict mortality of COVID-19 patients

**Fig 2:**
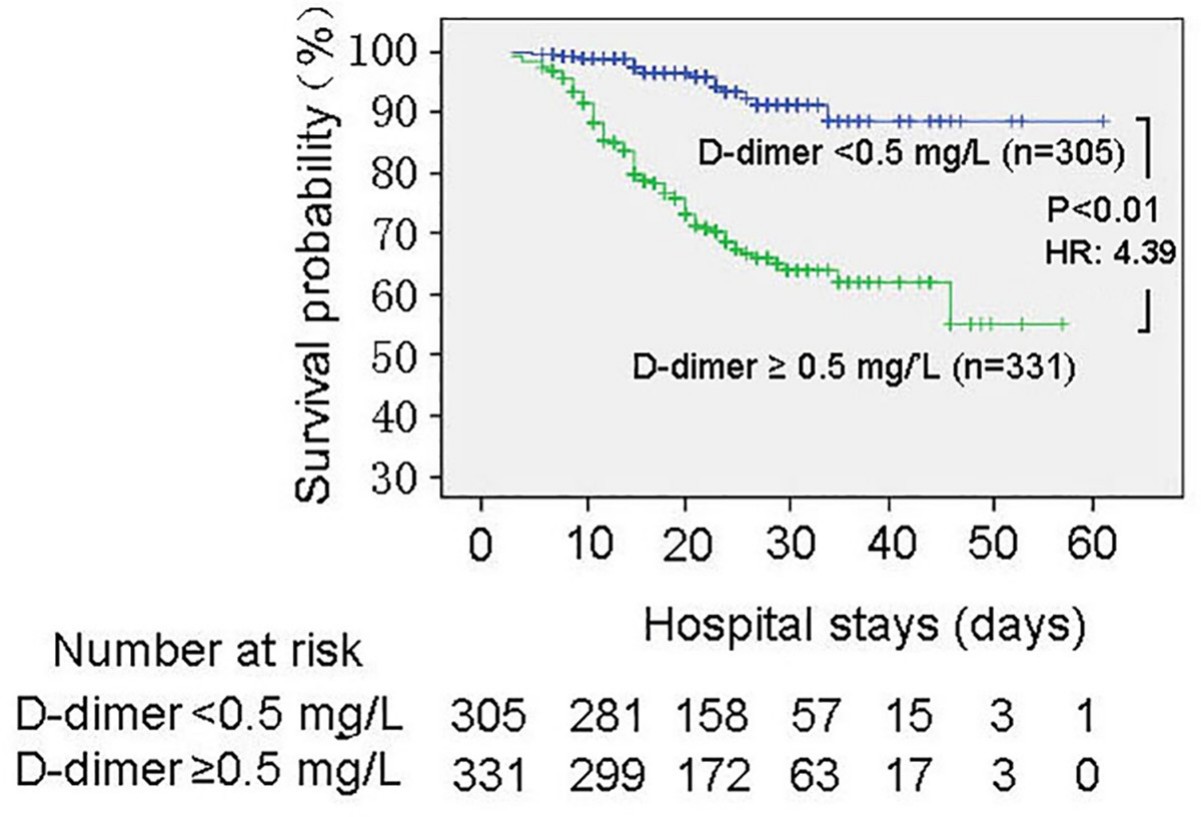
Kaplan-Meier survival curves for D-dimer levels on the initial admission. Statistic significance of separation between two groups was achieved at 8 days after admission. HR: Hazard ratio.

**Fig 3:**
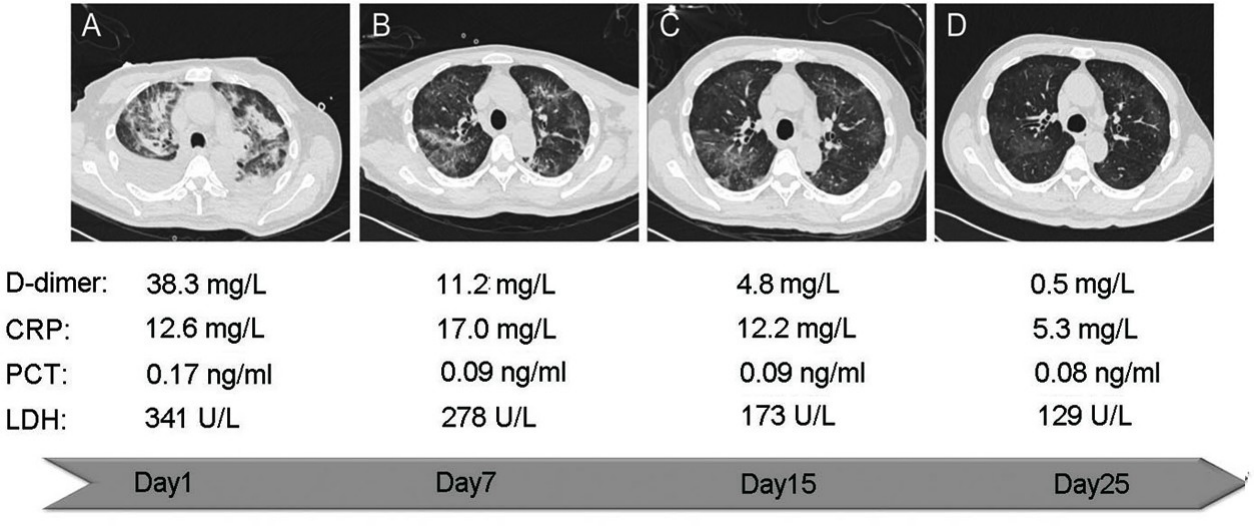
Representative computed tomography of a 62-year-old male patient during adjuvant antithrombotic treatment. Abidol and Ibuprofen was taken orally by the patient intermittently. After admission, the combination of anticoagulation was used (Aspirin enteric-coated tablets 100mg once a day and Clopidogrel bisulfate tablet 75mg once a day). The representative CT and the blood test results during the treatment were shown as A-D. (A) The infection was severe, and the diffuse distribution of the infection foci was dominated by bilateral pleural effusion. The D-dimer level was very high (38.3 mg/L). (B) The infection foci of the bilateral upper lung were reduced, and bilateral pleural effusion had been absorbed by itself. (C) The foci were reduced continuously and were mainly in the right upper lung at this time. (D) The infection foci were almost completely absorbed, while the D-dimer level of patient close to normal (0.5 mg/L).

Then, we further analyzed the role of D-dimer in predicting mortality of COVID-19. We found D-dimer has the highest C-index to predict the mortality compared with CRP, PCT and LDH after the admission (**Table 2**). Kaplan-Meier Survival Curves (**Fig 2**) showed that D-dimer level on the initial admission was the significant predictor of mortality (D-dimer ≥ 0.5 mg/L, HR: 4.39, *P*<0.01) (**Table 3**). Cox proportional hazard analysis showed that high D-dimer level was also a significant determinant (D-dimer ≥ 0.5 mg/L, adjusted HR: 1.75, *P =* 0.015) after adjustment of gender, age, underlying disease (Hypertension, Diabetes, Heart Disease and Cancer) and other risk factors in the blood (CRP, PCT and LDH), which means D-dimer is the independent predictor of COVID-19 mortality. Furthermore, we found the D-dimer levels were related with the status of patients and the severity of lung lesion through radiologic assessments. There are more events of severe lung lesion happened in the group which D-dimer expressed higher than 0.5 mg/L (37.3% vs. 18.1%). The representative images of chest computed tomography **(CT)** from a cured severe patient during the treatment was shown as **Fig 3**.

**Table 2:** C-index analysis of the clinical risk factors to predict the mortality of COVID-19.

C-index(95%CI)	Onset of admission	After admission	
Risk factors		7 Days	15 Days
D-Dimer	0.782 (0.736-0.828)	0.883 (0.835-0.929)	0.898 (0.832-0.954)
CRP	0.845 (0.807-0.883)	0.866 (0.825-0.908)	0.854 (0.809-0.920)
PCT	0.839 (0.797-0.881)	0.800 (0.741-0.864)	0.663 (0.592-0.733)
LDH	0.757 (0.700-0.815)	0.721 (0.663-0.779)	0.814 (0.761-0.866)
WBC	0.680 (0.622-0.738)	0.706 (0.642-0.771)	0.754 (0.696-0.812)
Neutrophil	0.720 (0.665-0.775)	0.740 (0.676-0.804)	0.780 (0.722-0.837)
Lymphocyte	0.674(0.596-0.747)	0.739 (0.683-0.794)	0.718 (0.655-0.782)
Platelet	0.678 (0.609-0.747)	0.723 (0.660-0.786)	0.674 (0.578-0.769)
Haemoglobin	0.587 (0.508-0.666)	0.503 (0.412-0.583)	0.532 (0.429-0.635)
CK	0.635 (0.569-0.706)	0.654 (0.570-0.738)	0.529 (0.433-0.626)
AST	0.653 (0.584-0.722)	0.625 (0.537-0.712)	0.676 (0.574-0.779)

Abbreviations: D-dimer, Dimerized plasmin fragment D; CRP, C-reactive protein; PCT, procalcitonin; LDH, lactate dehydrogenase; WBC, white blood cell; CK, creatinine kinase; AST, aspartate aminotransferase; CI, confidential interval.

**Table 3:** Univariate and multivariate Cox proportional hazard analysis of risk variables for hospital mortality.

Variables	Unadjusted		Adjusted	
	HR(95%CI)	P value	HR(95%CI)	P value
Age≥60	2.82 (1.94–4.11)	<0.01	1.62 (1.02–2.58)	0.041
Male gender	1.05 (1.04–1.06)	<0.01	1.03 (1.01–1.04)	<0.01
Hypertension	3.06 (2.15–4.37)	<0.01	1.90 (1.19–3.03)	<0.01
Diabetes	1.19 (0.77–1.83)	0.436	0.84 (0.53–1.28)	0.276
Heart Disease	1.65 (1.06–2.57)	0.027	1.40 (0.76–2.47)	0.297
Cancer	1.99 (1.10–3.60)	0.024	0.92 (0.66–1.44)	0.347
D-Dimer ≥0.5 mg/L	4.39 (3.01–6.39)	<0.01	1.75 (1.11–2.76)	0.015
CRP ≥10 mg/L	1.42 (1.22–1.62)	<0.01	1.21 (1.06–1.32)	<0.01
PCT≥ 0.5 ng/ml	1.24 (1.17–1.31)	<0.01	1.10 (1.01–1.19)	0.021
LDH ≥ 250 U/L	1.04 (1.03–1.04)	<0.01	1.01 (1.01–1.02)	<0.01

## 4. Discussion

The pandemic infectious disease COVID-19 caused by SARS-CoV-2 is rapidly spreading across the globe. Phylogenetic studies indicated that it is closely related to a bat-derived SARS-like coronaviruses [2], and belongs to a large Coronavirus family including severe acute respiratory syndrome coronavirus (SARS) [21] and Middle East Respiratory Syndrome (MERS) [22]. However, it is reported that COVID-19 has higher infection rates compared with SARS and MERS [21]. The early daily reproduction number (R_t_) of COVID-19 in Wuhan declined from 2.35 (95% CI, 1.15–4.77) [23]. It is still an emerging, rapidly evolving situation [24–28].

According to the WHO’s report, the recent mortality in the COVID-19 patients is about4% [1], and the epidemiological data suggest that the pandemic will continue at least for years. Though a number of the general epidemiological and clinical features of COVID-19 have been reported [4, 5, 29–31], the knowledge about the difference between survival and non-survival COVID-19 patients has not been comprehensively established, and the information of clinical markers for predicting the mortality of COVID-19 is still lacking. Thus, the aim of this cohort study is to elaborate the different clinical features between survival and non-survival COVID-19 patients, and find the optimal clinical risk factors for predicting the mortality of COVID-19.

In our cohort, we divided COVID-19 patients into survival and non-survival subgroups according to endpoint. Our study showed the mortality of COVID-19 was higher in elder people, male and the patients with underlying disease. Fever and cough were the dominant symptoms whereas gastrointestinal symptoms were rare. In the radiologic assessments, more events of severe lung lesions were found in the non-survival group. For the diagnosis markers, we found the D-dimer, CRP, LDH, PCT, and Interleukin-6 were increased significantly in non-survival group compared with the survival group on the initial admission, while ASP, ALP, CK and creatinine were also moderately increased. This means more inflammation, thrombus, myocardial injury or liver and kidney damage existed in non-survivors. Moreover, we studied the dynamics during the process of COVID-19 by timeline tracking. We found CRP, LDH, PCT had no obvious changes in non-survivors after admission, while D-dimer still increased quickly in some non-survivors and was most likely related with the mortality.

It is noticed that another two recent cohort studies [18, 19] and one meta-anlaysis [32] also showed that D-dimer was one of the risk factors for predicting the mortality over time in COVID-19. However, the previous cohort studies did not compare the difference between D-dimer and other markers comprehensively and did not further explore its role in the prognosis and therapy, and the authors considered the interpretation of their findings might be limited by the sample size of the cohort [18, 19]. In our study, we verified the role of D-dimer in predicting mortality by both C-index analysis and Kaplan-Meier survival analysis, based on a larger scale of sample size (676 cases). Furthermore, we found that antithrombotic therapy effectively improved the treatment in some patients, which is consistent with another report [33]. Indeed, when there is serious hypoxemia and hypotension in COVID-19 patients, the possibility of embolism should be considered, coagulation screening by D-dimer is suggested and the COVID-19-associated coagulopathy should be managed as it would be for any critically ill patient [33, 34]. Thus, we propose that severe patients of COVID-19 can be given preventive anticoagulants or physical therapy. Our study still has several limitations. Firstly, it is reported that some patients with positive chest CT findings may have negative RT-PCR results. In that case, there may be selection bias since only the positive cases confirmed by the RT-PCR were included in our study. Secondly, some COVID-19 patients were discharged, died or in unstable physical condition in the process. Although we tried our best to collect all the data from each blood sample and other clinical tests of patients, we could not acquire all the information of some patients at each time point designed, which may result in a considerable standard error of the time axis.

## 5. Conclusions

In summary, our study showed CRP, D-dimer, LDH, PCT are the high risk factors related with COVID-19. We found D-dimer was the best laboratory marker in our study. So far, there is no valid vaccine or specific medicine for COVID-19. As high mortality in the non-survival COVID-19 patients need to be resolved immediately, the risk factors explored in our study are important of taking into account the disease management in practice. Though the prognostic factors in our study still need to be further validated by future studies, they should be helpful to provide a real-time warning model for predicting mortality in COVID-19, which is very useful for clinical application.

## Abbreviations

IQR: interquartile range
PaO2:FIO2 ratio: partial pressure of arterial oxygen to the fraction of inspired oxygen
WBC: white blood cell
D-dimer: Dimerized plasmin fragment D
CRP: C-reactive protein
PCT: procalcitonin
LDH: lactate dehydrogenase
AST: aspartate aminotransferase
ALT: alamine aminotransferase
CK: creatinine kinase
